# Incorporating LGBT+ mental health into psychiatric residency and training: perspectives from the Philippines

**DOI:** 10.1192/bji.2023.39

**Published:** 2024-05

**Authors:** Rowalt Alibudbud

**Affiliations:** Faculty member, Department of Sociology and Behavioral Sciences, College of Liberal Arts, De La Salle University, Manila, Philippines. Email: rowalt.alibudbud@dlsu.edu.ph

**Keywords:** Sexual and gender minorities, mental health, medical residency, psychiatry, health professions education

## Abstract

The Philippine Mental Health Act upholds the rights and inclusivity of all people, regardless of gender and sexual orientation, within mental health services and programmes. Nevertheless, a noteworthy challenge lies in the inadequate attention given to the needs of LGBT+ individuals within the medical and health professions education in the country. Therefore, it is imperative to integrate LGBT+ mental health into psychiatric residency training. To address this gap, this paper proposes the inclusion of concepts relevant to LGBT+ mental health, including minority stress, intersectionality, identity concealment and LGBT+-affirming practices, to enhance the understanding and response to the needs of LGBT+ Filipinos.

In 2020, the Philippines achieved the highest ranking in Asia on the gender gap index established by the World Economic Forum, a testament to the country's constitutional commitment to gender equality. This commitment has spurred policy reforms aimed at improving the rights and protection of women, resulting in increased political participation, expanded educational opportunities and reduced discrimination for women.^1^ Despite these advancements, the lesbian, gay, bisexual, transgender and other sexual and gender minority (LGBT+) community in the Philippines continues to endure abuse, discrimination and prejudice stemming from their gender and sexual orientation.^2–9^

## LGBT+ mental health services and training in the Philippines

Discrimination and prejudice against LGBT+ individuals contribute to the elevated prevalence of mental disorders within this population.^8,10^ Disturbingly, more than a quarter of the Philippine population exhibits negative attitudes toward gays and lesbians.^6^ Moreover, there are numerous documented instances of discrimination against bisexual and transgender individuals.^7^ These distressing realities underscore the potential adverse impact of Philippine society on the mental well-being of LGBT+ Filipinos. Therefore, there is a compelling imperative to establish measures that safeguard and promote mental health among this marginalised population.

In 2018, the Philippines enacted its first Mental Health Act, featuring provisions designed to advance the rights and inclusivity of minoritised sexualities and gender identities within psychiatric and mental health services and programmes. This legislative inclusion is in response to the discernible discrimination against LGBT+ Filipinos within the mental health profession. For instance, a troubling episode in a 2011 Filipino national television programme showcased a mental health professional advising parents of LGBT+ children to employ conversion therapy as a means of achieving a ‘happy family life’, notwithstanding the therapy's well-documented detrimental consequences on mental health.^5,11^ Although several Filipino psychologists have taken the initiative to develop courses and practices addressing the mental health needs of LGBT+ people,^5^ challenges in LGBT+ mental healthcare are still apparent. These challenges manifest in the underrepresentation of LGBT+ needs in the curricula of the country's medical and health professions education, such as the limited training on gender-affirming care and the stigmatisation of transgender identities.^2^ Hence, there is an urgent necessity to implement measures that uphold gender-affirmative and inclusive psychiatric services and training in the Philippines.

In the Philippines, medical graduates who have successfully passed the Philippine Medical Licensure examination can apply for admission to hospitals accredited by the Philippine Psychiatric Association. These hospitals offer 3- to 4-year residency training programmes, leading to the board certification of psychiatrists in the Philippines. These programmes provide residents with comprehensive exposure to general psychiatry and the different psychiatric subspecialties, such as child and adolescent psychiatry and community psychiatry. Graduates of these residency training programmes are then eligible to proceed to the annual written and oral examinations administered by the Specialty Board of Philippine Psychiatry. Successful candidates are subsequently conferred the status of diplomate, representing board certification for psychiatrists practising in the Philippines.

Given the imperative to incorporate LGBT+ mental healthcare into psychiatric residency training in the Philippines, this paper proposes several key concepts that could be integrated into that training to advance LGBT+ mental health. Emerging evidence suggests the potential mental health benefits of this increased support and acceptance within the LGBT+ community in the country.^5,11^ Similar to LGBT+ mental healthcare training in other regions,^12–14^ these proposed concepts can be integrated into Philippine psychiatry residency programmes through mentorship, didactic sessions, clinical rotations and online training.

## LGBT+ mental health concepts for psychiatric residency training

### Minority stress

Psychiatric residency training could integrate the minority stress model into its programmes.^12^ The minority stress model provides a framework for understanding the mental health disparities experienced by LGBT+ individuals.^10^ Within this model, it is posited that unique social experiences account for the higher prevalence of mental disorders among LGBT+ individuals.^10^ These stressors are categorised into distal stressors, representing adverse social experiences rooted in one's sexuality and gender (e.g. discrimination and prejudice), and proximal stressors, encompassing cognitive processes that uniquely affect LGBT+ individuals (e.g. internalised homophobia).^10^ This model is applicable in the Philippine context, where research consistently highlights the negative impact of discrimination on the mental health of LGBT+ Filipinos.^3,4,8^ Therefore, incorporating the minority stress model into psychiatric residency training could provide trainees with essential insights into a pivotal factor perpetuating mental health disparities within the LGBT+ population. Notably, previous training models for psychiatric trainees exemplify the feasibility of incorporating this concept through modular training, mentorship and clinical experiences.^12–14^

### Intersectionality

Another concept that could be included in psychiatric residency training is the theory of intersectionality, an analytical framework developed by Crenshaw. Intersectionality considers different social positions, such as race, gender and sexuality, in elucidating the dynamics of privilege and discrimination within society.^15^ It has been applied to LGBT+ mental health in understanding how the confluence of different marginalised and privileged social positions affects mental health.^15^ Intersectionality is an important factor influencing LGBT+ mental health in the Philippines. For instance, variables such as age and romantic relationships have positively and negatively influenced the mental health of LGBT+ Filipinos.^3,4^ Therefore, the incorporation of intersectionality into psychiatric residency training could provide a deeper understanding of the intricacies and factors that underlie the mental health of LGBT+ Filipinos. In practice, this inclusion could serve as a framework for understanding the social factors involved in psychiatric case formulations and patient presentations.

### Identity concealment

The Philippines has not enacted the Sexual Orientation and Gender Identity or Expression (SOGIE) equality bill, a legislative endeavour designed to protect Filipinos from violence, abuse and discrimination arising from their SOGIE.^3^ Therefore, LGBT+ Filipinos may hide their SOGIE-based identities to avoid societal marginalisation and discrimination.^3,7,9^ In this regard, psychiatrists and other mental health professionals should understand the effects of SOGIE-based identity concealment on the mental health of LGBT+ Filipinos. For instance, a review conducted by Pachankis et al^16^ shows a positive association between sexual orientation concealment and mental health problems, including depression, anxiety and distress. Therefore, the inclusion of identity concealment in existing training programmes could sensitise future Filipino psychiatrists to the need to assess SOGIE and its added complexities associated with the mental health of LGBT+ Filipinos. Consistent with prior initiatives in the field of LGBT+ mental health training, the relationship between identity-related concerns and mental health could be incorporated into didactic and clinical experiences (e.g. journal clubs or patient case presentations) of psychiatric trainees.^12^

### LGBT+-affirming practices

The interaction between mental health clinicians and their patients serves a dual purpose, encompassing clinical assessment and management.^17^ Beyond the explicit objectives, this relationship is a therapeutic encounter that can benefit mental health.^17^ Therefore, it is necessary to foster affirmation and inclusivity, as well as avoid prejudice and discrimination, in psychiatric and mental health services, particularly among LGBT+ people. This could be done by integrating the biological and psychosocial aspects of LGBT+-affirming practices.

Emerging evidence of gender-affirming biologically based interventions and their effects on mental health could be incorporated into existing training topics, such as gender dysphoria.^2^ For instance, the large prospective study conducted by Chen et al^18^ revealed that gender-affirming hormones improved appearance congruence and psychosocial functioning and decreased depression and anxiety symptoms.

Discussions within training programmes should also encompass the psychosocial dimensions of gender-affirming practices. For instance, international guidelines on gender-affirming mental healthcare, such as the utilisation of preferred names and pronouns to foster comfort and ease among LGBT+ individuals,^19^ could be adapted and culturally validated in the Philippine setting.

By incorporating the biological and psychosocial aspects of LGBT+-affirming practices into existing psychiatric residency and training, future generations of psychiatrists could be better equipped to assess, address and analyse the multifaceted needs of LGBT+ Filipinos. As a start, training programmes could culturally adapt and incorporate evidence-based training methods, such as in LGBTQ-affirmative cognitive–behavioural therapy.^14^ This training encompasses discussions of socially relevant factors, such as minority stress, and the practical implementation of LGBT+-affirmative practices, including behavioural skills training. This approach could significantly enhance the competence of future psychiatrists in providing affirming care to LGBT+ individuals in the Philippines.

## Conclusions

The Philippine Mental Health Act outlines provisions upholding the rights and inclusion of minoritised sexualities and gender identities in psychiatric and mental health services. However, the invisibility of LGBT+ needs in medical and health professions education presents a challenge. To address this, it is essential to integrate LGBT+ mental health into psychiatric residency and training programmes. This can commence by culturally adapting established training practices and modifying existing curricula to incorporate key concepts such as minority stress, intersectionality, identity concealment and LGBT+-affirming practices. Support for this initiative could be enhanced by increasing research funding for LGBT+ mental health, improving social services for marginalised LGBT+ populations and expanding LGBT+ mental healthcare training programmes for allied health services. By implementing these measures, the Philippines could strive towards a more inclusive and responsive mental health system equipped to address the needs of LGBT+ individuals.

## Data Availability

Data availability is not applicable to this article as no new data were created or analysed in this study.
